# Why is rumination unhelpful in adolescents? Two studies examining the causal role of abstract processing

**DOI:** 10.1016/j.jad.2025.03.058

**Published:** 2025-03-11

**Authors:** Eleanor Leigh, Lorna Taylor, Victoria Cole, Patrick Smith

**Affiliations:** Department of Experimental Psychology, University of Oxford; Department of Psychology, Institute of Psychiatry Psychology & Neuroscience, King’s College London, London SE5 8AF, UK

## Introduction

Given the rise in the incidence of depression during adolescence (Shorey et al., 2022) and the long-term individual and economic burden of the disorder (Greenberg et al., 2023), improving treatment outcomes is a priority. Although their use is widespread, cognitive and behavioural interventions for youth depression are associated with only modest effects (Eckshtain et al., 2020) and outcomes are worse for young people compared to adults (Cuijpers et al., 2020). Using experimental studies to test the causal relationship between putative psychological maintenance factors and symptoms is an effective research strategy for improving treatment effects. One psychological process that has been implicated in both adult and adolescent depression is rumination (Abela & Hankin, 2011; Nolen-Hoeksema et al., 2008).

Rumination, defined as a tendency to respond to low mood with a negative, abstract, self-focused and repetitive style of thinking, is characteristic of depression (Smith & Alloy, 2009). Amongst dysphoric and depressed adults, experimental studies have demonstrated that compared to distraction, engaging in rumination is associated with lowered mood and adversely affects a range of processes known to be risk factors in depression, including social problem-solving (Lyubomirsky & Nolen-Hoeksema, 1995) and negative future thinking (Lavender & Watkins, 2004). According to the “processing mode hypothesis” detrimental effects of rumination are, at least in part, caused by the abstract processing mode that is characteristic of rumination (Watkins, 2008; Watkins & Roberts, 2020). It is suggested that the high-level mental representations in abstract thinking are somewhat decontextualized (conveying the ‘gist’) and entail generalised inferences and evaluations (Watkins, 2008; Watkins et al., 2015). Abstract construals are typically concerned with the desirability and importance of outcomes or goals. In contrast, lower-level construals, associated with the concrete mode, are detailed, specific, and concerned with the particulars of a given situation. Concrete construals are focused on the planning and implementation of outcomes. One’s own and others’ behaviour can be construed at varying levels of abstraction, and it is suggested that processing negative material at an abstract level is unhelpful (Watkins, 2008). A series of studies undertaken by Watkins and colleagues (Watkins, 2008) with adults compared the effects of an abstract, verbal-analytical and evaluative processing mode, consistent with the ruminative mindset, with a concrete, experiential mode of processing, with a focus on the here and now and current feelings, that stands in contrast to the ruminative mindset. Although there are some exceptions (e.g., (Mogoaşe et al., 2013)), the weight of studies have found that amongst dysphoric (high symptoms) and depressed (as established by clinical diagnosis) adults, abstract processing was associated with poorer social problem-solving (Watkins & Baracaia, 2002), more overgeneral memory (Watkins & Teasdale, 2001), and greater emotional reactivity (Watkins et al., 2008) compared to concrete processing. Taken together, the studies are consistent with the suggestion that processing mode moderates the effects of rumination.

Turning to adolescents, an association between rumination and depressive symptoms has been reported in non-clinical populations of young people during middle childhood (Ziegert & Kistner, 2002) and early adolescence (Abela et al., 2004) and the propensity to ruminate has been shown to be associated with increased likelihood of onset of a major depressive episode and duration of episodes in adolescents (Abela & Hankin, 2011). A qualitative study indicated that clinic-referred young people with depression experience rumination often starting with a specific trigger but quickly progressing to global and abstract thoughts (Oliver et al., 2015). Experimental studies indicate that, compared to *distraction*, ruminative processing exacerbates negative affect (Rood et al., 2012) and cognitive factors implicated in depression such as overgeneral autobiographical memory (Park et al., 2004). As yet, whilst the processing mode hypothesis has been examined with student samples (Kambara et al., 2023), it not been tested experimentally in younger adolescents.

Here, we describe two experiments that aim to test the causal role of processing mode in the maladaptive effects of rumination in adolescents. Specifically, we test the hypothesis that processing mode causally influences two processes known to be important in adolescent depression: social problem-solving and negative future thinking.

**Study 1** examines the effects of manipulating processing mode on social problem-solving. Social problem-solving is a known risk factor for depression in adults (Nezu, 1987) but it may be even more important during adolescence because this is a time when peer relationships are especially salient. Correlational studies suggest self-reported poor social problem-solving is associated with depressive and suicidal symptoms in adolescents (Asarnow et al., 1987; Fremouw et al., 1993; Rotheram-Borus et al., 1990; Sadowski & Kelley, 1993; Spirito et al., 1989). Using an objective experimental measure of social problem-solving, the Means End Problem Solving task (MEPS), adolescents with depression generated less effective solutions compared to healthy and to anxious control groups (Marx et al., 1992).

Given that the first step in effective problem-solving involves problem specification (D’Zurilla & Nezu, 1982) it seems plausible that processing information in an abstract style, for example, thinking about ‘why’ the problem happened in a general and abstract way, and the implications of the problem, will be unhelpful. In line with this, the detrimental effects of abstract, compared to concrete processing on social problem-solving has already been observed in *adults* (Sanders & Lam, 2010; Watkins & Baracaia, 2002) and related constructs of decision-making (Dey et al., 2018) and proactivity (Dey et al., 2019). As yet, no studies have been undertaken investigating the effect of processing mode on social problem-solving in adolescents. Here, we test the hypothesis that engaging in abstract processing will impair social problem-solving compared to engaging in concrete processing in adolescents scoring high but not low in depression symptoms (i.e. we hypothesized we would find an interaction between depression group and processing mode condition on social problem-solving). In order to enhance the ecological validity of the findings by linking the experimental results to real-world patterns, we were also interested to test the hypothesis that we would observe a negative correlation with performance during abstract processing but not concrete processing.

**Study 2** examines the effects of manipulating processing mode on future thinking in adolescents. Hopelessness, defined as pessimistic thinking and expectation for the future, is a fundamental component of depression, including in adolescents (Lakshmi et al., 2024). Ruminative thinking has been shown to exacerbate hopelessness in adults. For example, dysphoric adults who have engaged in analytical rumination exhibit priming for hopeless cognitions (Nolen-Hoeksema, 1991) and demonstrate a lower expectation of future positive events (Lyubomirsky & Nolen-Hoeksema, 1995) compared to those who have engaged in a period of distraction. Lavender and Watkins (2004) reported similar findings for clinically depressed adults: participants who engaged in a period of ruminative processing demonstrated increased negative future thinking compared to those who had engaged in a distraction task. The effects of manipulating processing mode on future thinking has not been examined in adults.

Hopelessness is a cardinal symptom in adolescent depression also. For example, dysphoric adolescents show increased negative thinking about the future compared to euthymic adolescents (Miles et al., 2004). Among adolescents referred to mental health services, there is a direct association between the severity of depressive symptoms and the extent of pessimistic thinking about the future, independent of anxiety (Muris & van der Heiden, 2006). The long-established association between hopelessness and parasuicidal behaviour in adolescents with depression (Abramson et al., 1998; Joiner & Rudd, 1996) underlines the importance of future expectation in depression. With Study 2, we aim to test the hypothesis that amongst adolescents with high levels of depressive symptoms, engaging in abstract processing will generate more negative future thoughts compared to engaging in concrete processing, but we would not observe this effect in adolescents low in depression symptoms. As in Study 1, we also aimed to test the hypothesis that we would observe a negative correlation with future thinking during abstract processing but not concrete processing.

We undertook Studies 1 and 2 with a school-based sample of adolescents scoring in the upper and lower quartile of a measure of depression symptoms. This is in line with the dimensional approach to psychopathology advocated by the National Institute of Mental Health Research Domain Criteria (Garvey et al., 2016), and the proposal of Woody & Gibb (2015) in relation to depression research to study specific processes such as rumination across the full range of functioning, not just within the diagnostic taxonomy. We chose to use a quartile split because we were recruiting a non-clinical population from schools. Clinical cutoffs are typically designed for diagnostic or clinical populations and may not be sensitive to the distribution of symptoms in a general school population, where symptom levels might be lower or more variable. Quartile splits capture the variation within the general population, allowing a clearer distinction between the extremes (high and low symptom groups).

## Methods

### Ethical Approvals

Ethical approval for the study was granted from King’s College London Psychiatry, Nursing and Midwifery Research Ethics Sub-committee (Ref: PNM/10/11-148).

### Design

Young people scoring high and low on a measure of depressive symptoms were randomly allocated to take part in either Study 1 or Study 2. Within Study 1 and Study 2, high and low participants were then randomly allocated to either the Abstract or Concrete Thinking condition. Each condition comprised two steps: first, a thinking style training phase during which participants received training in abstract or concrete thinking (depending on the condition); second, a testing phase, when participants were asked to continue thinking in the designated thinking style whilst completing a social problem-solving task (Study 1) or a future thinking measure (Study 2).

### Inclusion and Exclusion Criteria

Young people aged 11-16 years (years 7-10) from a large South London secondary school were screened on a measure of depressive symptoms (n = 497), the Mood and Feelings Questionnaire (MFQ; (Angold et al., 1995)). All eligible students in Years 7 to 10 (inclusive) were invited to participate in the screening which was undertaken in schooltime. Of the initial sample screened, 273 pupils were invited to participate in experimental studies (High MFQ: n = 135; Low MFQ: n = 138). Of those invited to participate, 42 (15.38%) did not consent (themselves or their parents) to take part (High MFQ: n = 23, Low MFQ: n = 19). Analysis revealed that pupils who opted out from the experimental tasks did not significantly differ from those who participated with regard to the presence of symptoms of depression or anxiety or propensity to ruminate at screening (as measured by the MFQ, Screen for Child Anxiety Related Emotional Disorders [SCARED] and Children’s Response Style Questionnaire [CRS-Q] respectively; full details of measures are provided below).

The remaining 231 were invited to take part in one of two experimental studies being run; those scoring in the upper quartile (calculated per school year group) were included in the High Symptoms group (n = 112) and those in the lower quartile (calculated per year group) were included in the Low Symptoms group (n = 119). These participants were then randomly allocated to one of two experimental studies, controlled to ensure an even weighting of high and low scorers from each year group in each study.

### Materials

#### Self-Report Questionnaires

*Demographics*, namely gender and age (in years and months) were collected by self-report.

*Mood and Feelings Questionnaire (MFQ-C)* is a 33-item questionnaire assessing symptoms of depression in young people (8-18 years) over a two-week period, ranging from 0 (not true) to 2 (often true; ranging from: 0-66). The measure has been show to demonstrate good internal consistency and adequate test – retest reliability (Angold & Costello, 1988; Wood et al., 1995).

*Child Response Style Questionnaire (CRSQ;* (Abela et al., 2002)*)* is a 25-item self-report questionnaire that assesses the tendency to respond to sad feelings with rumination in young people, across three subscales: Rumination, Distraction, and Problem-Solving. The Rumination subscale includes 13 items (total scores range: 0-39), with higher scores indicating a greater propensity to ruminate. The C-RSQ Ruminative Response subscale has moderate levels of internal consistency and exhibits good test- retest reliability (Hankin, 2008).

*Screen for Anxiety and Related Disorders (SCARED;* (Birmaher et al., 1997)*)* consists of 41 items related to anxiety rated on a score of 0 (not true) to 2 (often true) (total score ranging: 0-82) with higher scores indicating greater anxiety difficulties. The measure has been demonstrated as having good internal consistency, test-retest reliability, and discriminant validity, both within anxiety disorders and between anxiety and other psychiatric disorders (Birmaher et al., 1997).

*Attentional Control Scale – Children (ACS-C* (Derryberry & Reed, 2002)*)* is a 20-item measure of attentional ability (total scores ranging: 20-80), with higher scores reflecting greater attentional control. The measure has been demonstrated as having adequate internal consistency, construct validity and reliability (Muris et al., 2008).

*Hopelessness Scale Children (HSC;* (Kazdin et al., 1986)*)* is a 17-item designed to assess levels of hopelessness in young people and adolescents. Items are rated as ‘true’ or ‘false’ and total scores range from 0-17, with higher scores indicating a greater degree of hopelessness. Respondents are to answer each item based upon the past week. The scale has acceptable psychometric properties (Kazdin et al., 1986).

#### Experimental Procedures and Tasks

##### Means-End Problem-Solving Task (MEPS; (Platt & Spivack, 1975)) (Study 1 only)

The MEPS task measures participants’ ability to generate step-by-step means of achieving a goal. For each MEPS situation, participants are presented with the beginning and the end of a problem situation, and then asked to identify the steps they would take to reach the goal verbally (with answers recorded by the researcher). The MEPS has satisfactory internal consistency (from 0.80 to 0.84) and construct validity (e.g., Platt & Spivack, 1972). Consistent with previous studies (Lyubomirsky & Nolen-Hoeksema, 1995; Watkins & Baracaia, 2002) a shortened version was used, comprising four problems selected to specifically reflect social problems. Following feedback from piloting with adolescents, adaptations were made to ensure the scenarios were developmentally appropriate (e.g. Joffe (Joffe et al., 1990) et al, 1990; Lyubomirsky & Nolen-Hoeksema, 1995). The four items used in the current study related to: resolving an issue with friends; a family member; a teacher; and making friends in a new neighbourhood. For each MEPS item, total number of problem-solving steps (more steps indicates better social problem-solving) and effectiveness rating (based on a 7-point effectiveness scale; Watkins & Baracaia, 2002), were generated. A strategy was considered effective if it maximised positive and minimised negative short and long-term consequences (D’Zurilla & Goldfried, 1971). Total scores for these two measures were calculated across all task items. One of the authors (VC) coded all data. A second researcher who was also trained in the MEPS coding technique but blind to group or condition coded a 20% proportion of all data, with excellent agreement (steps taken *Kappa* = .92, *p* < .001, effectiveness *Kappa* = .95, *p* < .001).

##### The Future Thinking Task (Study 2 only)

During the Future Thinking Task (FTT; MacLeod (MacLeod et al., 1993) et al., 1993) participants are instructed to think of as many experiences as they can, occurring over three different time periods in the future; the next week, the next year, the next 5 to 10 years. The task involves two conditions, positive and negative, each consisting of three trials each lasting 60 seconds. The exact instructions given prior to each trial were in line with those reported by Lavender and Watkins (2004), ‘try to think of as many future positive experiences, things that you are looking forward to or thinking that you would enjoy that could happen in the next week/year/ 5 to 10 years’ for the positive trials and ‘try to think of as many future negative events, things that you are worried about or not looking forward to that could happen in the next week/year/ 5 to 10 years’. Answers were given verbally by participants and recorded by a researcher.

Within each condition, the trials were counterbalanced by block, such that half of the participants received the three positive trials and then the three negative trials and the other half completed the trials in the reverse order. The number of events generated in each time period and a total number of events generated across the time periods are summed to provide totals for positive and negative expected future events.

##### Verbal Fluency

Word Generation tasks from the NEPSY (Korkman, Kirk and Kemp, 1998 (Korkman et al., 1998)) were used as a baseline measure of verbal fluency. Participants completed semantic and initial letter word generation tasks, in which young people are asked to name as many items as possible in a particular semantic category or beginning with a certain letter in a 60 second period. This was included to control for possible group differences in verbal fluency that may account for any group differences found in social problem-solving ability or future thinking ability.

##### Thinking Style inductions

Following randomisation, participants were trained to engage in an abstract or concrete thinking style. Instructions were based on those outlined by (Watkins & Moberly, 2009). Minor adaptations (to wording and the example scenarios) were made following piloting with adolescents^1^.

In the abstract condition, participants were introduced to this type of thinking and shown a list of questions that characterize abstract processing, encouraging a focus on the causes, consequences and meaning of a situation. For example, “*why did this happen to me?*” For those in the concrete thinking condition, the type of thinking was explained to participants and they were shown a list of questions that characterize concrete thinking, encouraging a focus on the ‘here and now’ and direct experience of the situation, for example “*what did I notice?*”

The training procedure was otherwise comparable for the two conditions. Participants practiced thinking about a series of two imagined and two personal scenarios in the designated thinking style for two-minute periods (i.e. a total of 8 minutes training), with the aid of the written list of thinking style questions. Firstly they were presented with two imaginary scenarios (forgetting an important piece of school work, being unable to attend a friend’s party^2^). They were asked to imagine these scenarios as vividly as possible and to focus on associated thoughts, feelings and physical sensations. They were then instructed to continue thinking about the scenario in the designated thinking style, using the questions presented on the prompt card.

Participants then undertook the same practice exercise in relation to two personal real-life incidents that had occurred in the previous week and had made them feel sad. If needed, further feedback and explanation was given to ensure that participants understood the instructions.

##### Thinking Style Ratings to Assess Compliance

In order to assess participants’ ability to engage in the designated processing style, and as a measure of compliance with task demands, participants were asked to rate the proportion of time that they had been able to think in the mode instructed. End points of the scales were labelled ‘*not at all*’ and ‘*all of the time’*.

##### Mood Ratings

Mood (‘sadness’) ratings were recorded on 10cm Visual Analogue Scale (VAS) ranging from ‘not at all’ to ‘very’. Ratings were collected at seven time points to provide repeated-measures outcomes: at baseline; after each two minute thinking induction scenario during the processing mode induction; after completion of each experimental task. Participants indicated their current sadness level by marking a ‘X’ on each VAS. Change in mood was based on the first and last ratings in the session (i.e., before the processing mode induction and after the experimental task).

#### Procedure

Participants completed the MFQ depression measure and the CRSQ rumination subscale as part of the initial screening phase of the study (2-4 weeks prior to the experimental phase). To ensure stability of depression symptoms, participants completed the MFQ for a second time during the experimental session. They then completed the verbal fluency task. Baseline mood was assessed on VAS, and then participants were trained in the allocated thinking style. Thinking style ratings and state mood were assessed after each practice item. After the induction procedure, participants were asked to continue engaging in the indicated mentation style whilst they completed the experimental task (the MEPS in Study 1 and the Future Thinking Task in Study 2), before making final mood ratings. Participants were debriefed at the end of the experimental procedure.

#### Statistical Analysis

Following systematic checks for entry errors, a proportion (10%) of all data from each year group was double entered by a second researcher to check for data entry discrepancies and was found to be 100% consistent. No data was missing for the experimental sessions because this was overseen by a researcher. A small number of items were missing on questionnaires completed at screening. Where the measures included subscales, missing scores were replaced with individual subscale means from all other present scores for that subscale for that participant. Where total scores were used, missing scores were replaced with average scores of all other present scores for the individuals. Data were excluded in instances where more than 10% of items were missing (although this event did not occur).

All analyses were run in SPSS and R. ANOVA was used to compare groups and conditions on continuous baseline variables (age, MFQ, CRSQ, SCARED, ACS and HSC [for study 2 only]) and Chi square test for categorical variables (gender). ANOVA was also used to compare across groups and conditions for the following variables: compliance with instructions; emotional reactivity; MEPS steps and effectiveness (study 1 only); and order effects in the future thinking task (study 2 only). MANOVA was used to compare groups and conditions on the future thinking task (study 2 only). When controlling for mood reactivity, ANCOVA was used in analysis of MEPS scores (study 1 only) and MANCOVA for future thinking task scores (study 2 only). The associations between trait rumination (CRSQ) and MEPS scores (study 1) and future thinking (study 2) were examined with bivariate correlations. Results of Kolmogorov-Smirnov tests indicated the data for both Study 1 and Study 2 were normally distributed and there were no outliers. Results were examined to ensure that analyses had not violated the underlying assumptions of the test employed. For ANOVA this involved inspection of Levene’s test of equality of error variances to examined homogeneity of variances. ANCOVA analysis involves the same assumptions as ANOVA and two additional considerations 1) independence of covariate and experimental effect and 2) homogeneity of regression slopes. For MANCOVA this included assumptions of Multivariate normality and equality of covariance matrices (Box’s test). All assumptions were met.

## Study 1 Results

### Participant Characteristics

A total of 116 participants completed Study 1. Young people were excluded if the score they endorsed when the MFQ was repeated at the experimental testing session no longer fell within the quartile range for inclusion as calculated at screening. 21 participants were excluded for this reason.

In the final sample there were 95 participants, 50 participants in the High Symptoms group and 45 in the Low Symptoms group. The average age of the sample was 12 years 9 months (*SD* = 1.34) with no significant group, condition, or interaction effects on age (F<1 for all effects; descriptive statistics are shown in Table 1). There were 45 females and 50 males, with significantly more females in the High Symptoms group (n = 29) than the Low Symptoms group (n = 16; χ^2^=4.79, *p*=.03). There were no gender differences across condition. A series of ANOVA were run to compare groups and conditions across various measures. The same pattern of results emerged for all analyses with MFQ, SCARED, C-RSQ, and ACS: there was a main effect of group on depression symptoms (as indexed by the MFQ; *F*(1, 90) = 412.71, *p*<.001, partial *η*^*2*^ = 0.82); anxiety symptoms (as assessed by the SCARED; *F*(1, 89) = 123.82, *p*<.001, partial *η*^*2*^ =0.56); trait rumination (measured by the C-RSQ; *F*(1, 88) = 143.04, *p*<.001, partial *η*^*2*^ =0.62); and attentional control (measured by the ACS; *F*(1, 88) = 39.84, *p*<.001, partial *η*^*2*^ =0.31). Means and standard deviations are presented in Table 1. No other effects were significant (*p*>.05^3^). The ANOVA with verbal fluency revealed no significant main effects or interactions (*p*>.05).

### Compliance with Manipulation Instructions

ANOVA revealed that were no significant differences in the self-reported time spent thinking using the questions between high and low symptom groups or between conditions (Condition: *F*(1, 91) = 2.612, *p* = 0.251; Group: *F*(1, 91) = 4.578, *p* = 0.129; Group X Condition: *F*(1, 91) = 0.001, *p* = .995). Average compliance for the sample as a whole (on a scale from 0-10) was 6.93 [SD=1.40]. As a conservative test, all analyses reported below were rerun including only those participants endorsing at least 5/10 on the compliance scale (this resulted in the exclusion of n=18, Fisher’s exact test indicated there were no differences in the proportion excluded across groups and conditions). The results were unchanged and so analyses with the full sample are reported.

### Change in Mood

A main effect of Group was found for mood change across the session (*F*(1,81)= 4.114, *p* = .041, η2=.048), with greater overall increase in self-reported sadness in the high MFQ group [M=2.037] than in the low MFQ group [M=1.125]. No significant main effects of Condition (*p*=.074) or Condition x Group (*p*=.523) were found.

### MEPS performance in abstract and concrete thinking

Analyses were initially run with gender as a between subjects variable. No effects of gender were found on either outcome measure and therefore all analyses reported were conducted by collapsing across gender.

#### Total number of steps generated

**Figure 1:**
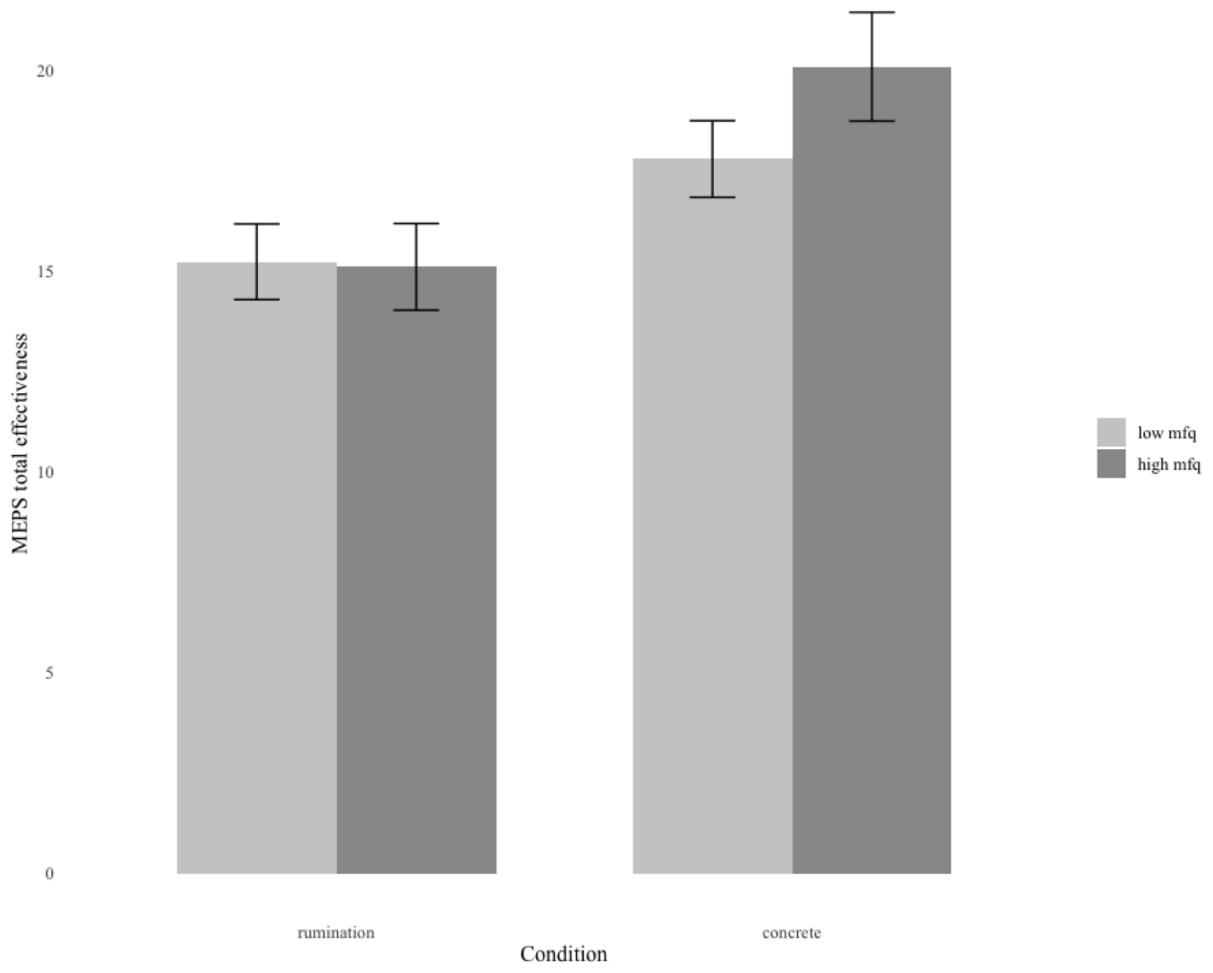
Total number of steps on the MEPS task across High and Low depression groups and the two Processing Mode Conditions.

A 2x2 between subjects ANOVA comparing the total number of steps generated in the concrete and abstract conditions by High and Low Depression group was undertaken. This revealed a significant main effect of condition on the total number of steps generated (*F*(1, 91) = 10.89, *p* = .001, partial *η*^*2*^ = .11), with a greater number of steps generated in the concrete thinking condition (*M* = 19.06 [SD=6.18]) compared to the abstract condition (*M* = 15.16 [SD=4.73]), as can be seen in Figure 1. No other effects were significant (Group: *F*(1, 91) = 0.91, *p* = .344; Group x Condition: *F*(1, 91) = 1.13, *p* = .291).

To investigate whether the observed difference was due to mood, an ANCOVA with changes in state mood as the covariate was conducted. The ANCOVA revealed that the main effect of condition on number of steps generated remained significant (*F*(1,87)= 6.46, *p* = .01, partial *η*^*2*^ = .07), with a greater number of steps generated in the concrete thinking condition compared to the abstract condition. This suggests that the observed difference in number of steps is unlikely to be due to changes in mood. No other effects were significant (Group: *F*(1, 87) = 0.78, *p* = .379; Group x Condition: *F*(1, 87) = 1.13, *p* = .291).

#### Effectiveness

**Figure 2:**
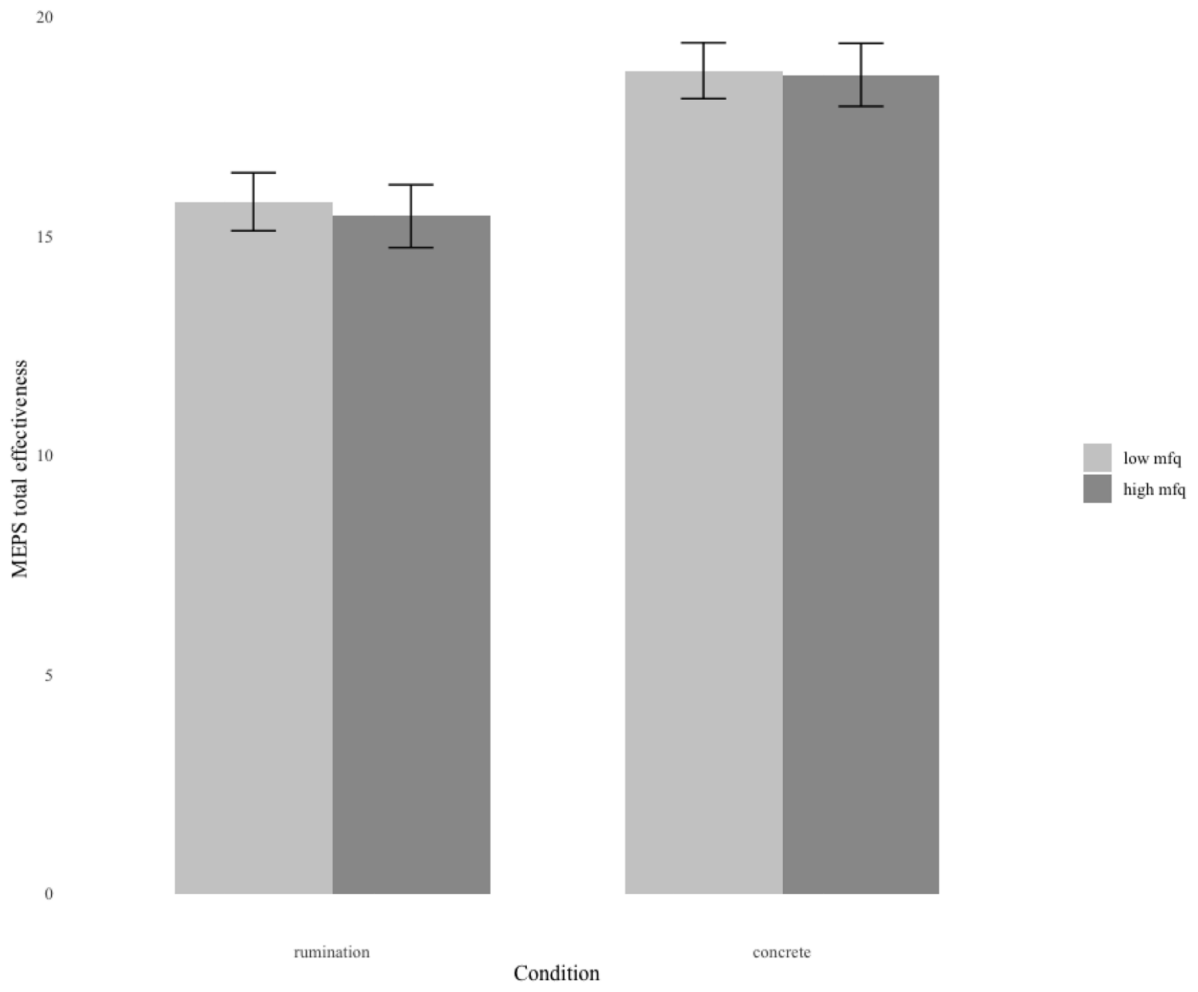
Total effectiveness score on the MEPS task across High and Low depression groups and the two Processing Mode Conditions.

A 2x2 between subjects ANOVA comparing the effectiveness of the solutions generated in the concrete and abstract conditions by High and Low Depression group was undertaken. This revealed a significant main effect of Condition (*F*(1, 81) = 19.92, *p* < .001, partial *η*^*2*^ = .18) with greater solution effectiveness in the concrete condition (*M* = 18.72 [SD=3.43]) compared to the abstract condition (*M* = 15.62 [SD=3.24]), as can be seen in Figure 2. No other effects were significant (Group: *F*(1, 91) = 0.09, *p* = .762; Group x Condition: *F*(1, 91) = 0.03, *p* = .867).

To investigate whether the observed difference was due to state mood, an ANCOVA was conducted with changes in state mood as the covariate. The previously observed main effect of Condition remained significant [*F*(1,87)= 11.28, *p* = .001, partial *η*^*2*^ = .12] with greater overall solution effectiveness in the concrete compared to the abstract condition. This suggests that the difference in total effectiveness is unlikely to be due to changes in mood. No other effects were significant (Group: *F*(1, 87) = 0.08, *p* = .785; Group x Condition: *F*(1, 87) = 0.06, *p* = .815).

#### Trait Rumination and MEPS Performance

Bivariate correlations were examined between self-reported trait rumination (the Rumination subscale of the CRSQ) and Total Number of Steps and Total Effectiveness Ratings in the Abstract and Concrete conditions. As expected, there was a significant negative association between scores on the Rumination subscale and Effectiveness in the Abstract Condition (*r* = -.373, *p* = 0.013) of medium magnitude. A medium negative association between trait rumination and Total Steps was found in the Abstract Condition, which approached significant (*r* = - 0.291, *p* = 0.056). Again, as expected, in the Concrete Condition, the associations between trait rumination and Effectiveness and Total Steps were small and not significant (*r* = -.111, *p*>0.05; and *r* = .105, *p*>0.05, respectively). Collapsing by condition, a small negative association was found at trend level between trait rumination and Effectiveness (*r* = -0.193, *p*= 0.065). The association between rumination and Number of Steps was small and not significant (*r* = -0.04, *r*>0.05).

## Study 1 Conclusions

For the first time in an adolescent population, our study has demonstrated that abstract thinking is associated with poorer social problem-solving compared to concrete thinking. Mood change did not account for the differences in social problem-solving between conditions. In contrast to hypotheses, social problem-solving was worse during abstract processing for both high and low depression symptom groups, with no group differences emerging in either condition.

## Study 2 Results

### Participant Characteristics

One hundred and fifteen participants completed Study 2. Data from 34 participants were excluded due to instability in their MFQ scores from screening to completion of the experimental stage (their scores at the experimental session no longer met criteria for inclusion in the quartile allocated at screening). The final sample consisted of 81 participants. There were 43 participants in the High group and 38 in the Low group. The average age of the sample was 12 years 9 months (*SD* = 1.26). Analyses revealed no significant differences in participants’ age across experimental Condition or Group (*p*>.05 for all effects). 43 participants were female. There were no gender differences across condition (χ^2^ = 2.72, *p* = .10), but there were significantly more females in the High group (n=29) than in the Low group (n = 11: χ^2^ = 7.58, *p* < 0.01).

As would be expected, ANOVA revealed a significant difference between the High (M=21.48 [SD=13.62]) and Low groups (M=1.95 [SD=2.11]) on MFQ scores (F(1, 77)=364.38, *p*<.001, η^2^=0.83). There were no significant differences on MFQ scores across experimental condition and no interaction effects (*p*>.05 for all effects). ANOVA’s revealed that the High group endorsed significantly higher levels of trait rumination than the Low group as measured by the rumination subscale of the C-RSQ (F(1, 76)=118.79, *p*<.001, η^2^=0.61), significantly higher anxiety levels (as measured by the SCARED; F(1, 76)=107.23, *p*<.001, η^2^=0.59), significantly poorer attentional control (as measured by the ACS; F(1, 74)=17.86, *p*<.001, η^2^=0.19) and significantly higher levels of hopelessness (as measured by the HSC; F(1, 74)=14.05, *p*<.001, η^2^=0.17). There were no significant condition or interaction effects (*p* > 0.05 for all). There were no differences across groups or conditions in verbal fluency of participants (p > 0.05 for all). Mean scores are shown in Table 2.

On the basis of previous research and the identification of verbal fluency as independent of the experimental groups and conditions (Lavender & Watkins, 2004), correlations between dependent variables and verbal fluency were examined to determine the inclusion of verbal fluency as a covariate in analyses. Verbal fluency was found to correlate with both positive and negative future thinking (p<0.05) and was included as a covariate in these analyses.

### Compliance with Instructions

ANOVA revealed no differences between groups or condition in compliance with instructions (Group: *F*(1, 77) = 2.949, *p* = .090, η2 = 0.037; Condition: *F*(1, 77) = 0.843, *p* = .361, η2 = 0.011; Group x Condition: *F*(1, 77) = 0.434, *p* = .512, η2 = 0.006). As a conservative test, all analyses reported below were rerun including only those participants endorsing at least 5/10 on the compliance scale (this resulted in excluding n=11, χ^2^ tests indicated there were no differences in the proportion excluded across groups and conditions). The results were unchanged and so analyses with the full sample are reported.

### Change in Mood

There was no significant main effect of Condition on change in sadness (*F*(1, 77) = 0.45, *p* = 0.503). There was a significant main effect of Group (*F*(1,77) = 7.295, *p* = 0.009, η2 = 0.123) with participants in the High MFQ group reporting a greater change in mood (Mean = 1.21, SD = 2.22) than participants in the Low MFQ group (Mean = 1.02, SD = 1.50). There was no significant interaction between Group x Condition (*F*(1,77) = 2.910, *p* = 0.094, η2 = 0.053).

### Future thinking

The order of presentation of negative and positive future thinking within the FTT was counterbalanced across participants. 2 x 2 ANOVA analysis revealed a significant main effect of presentation order on the number of positive future items generated (F(1,75) = 10.88, p<0.001, η^2^ = 0.127), with participants generating positive future events first generating a higher number of total positive events (Mean = 15.84, SD = 4) than those required to generate negative future events first (Mean = 11.68, SD = 4.36). Presentation order was entered as a covariate in analysis of positive future thinking. There was no significant effects of presentation on the number of negative future items generated (F(1, 75)=0.13, *p*=721).

#### Negative future thinking

2 x 2 MANCOVA analyses, with verbal fluency entered as a covariate, revealed a significant effect of Condition on the total number of negative future events generated, F(3,74) = 7.21, p<.001, partial η^2^ =0.23, with participants reporting a higher number of negative future events during abstract processing than concrete processing. There was significant effect of Group (F(3,74) = 3.87, p=.012, partial η^2^ =0.14), with High scorers reporting more negative future events. There was no Group x Condition interaction effect. Univariate ANCOVA’s revealed significant effects of Condition on the number of negative future events generated across each of the three time periods; the next week (F(1,76) = 14.72, p<0.001, η^2^= 0.16), year (F(1,76)= 11.37, p<0.001, η^2^=0.13) and 5-10 year time periods (F(1,76) = 16.45, p<0.001, η^2^=0.18). Univariate ANCOVA’s revealed a significant effect of Group on the number of negative future events generated across the next week only (F(1,76) = 11.38, p<0.001, η^2^= 0.13).

A 2 x 2 MANCOVA, with changes in state mood entered as an additional covariate, revealed that the observed effect of Condition on negative future thinking remained significant with participants in the abstract processing condition generating significantly more negative future events than participants in the concrete processing condition (F(3, 75) =7.32, p<0.001, η^2^ =0.23). Similarly the main effect of group remained (F(3, 75) =4.02, p=.012, η^2^ =0.14)

#### Positive future thinking

A 2 x 2 MANCOVA, covarying for verbal fluency and presentation order, revealed no significant effect of Condition (*F*(3, 72) = 1.60, *p*=.197) or Group (*F*(3, 72) = 0.79, *p* = .502) on the number of positive future events generated. There was no significant interaction (*F*(3,72) = 1.062, *p* =.371). Analysis, covarying for verbal fluency, presentation order and also change in sadness, also revealed no significant effect of Condition (*F*(3, 71) = 1.37, *p*=.258) or Group (*F*(3, 71) = 0.80, *p*=.497) on the number of positive future events generated and no interaction effect (*F*(3, 71) = 0.92, *p*=.435). Descriptive statistics are shown in Table 3.

#### Trait Rumination and Future Thinking

Bivariate correlations were examined between self-reported trait rumination (the Rumination subscale of the CRSQ) and Future Positive and Negative Thinking in the Abstract and Concrete conditions. As expected, there was a significant association between scores on the Rumination subscale and Future Negative Thinking ni the Abstract Condition (*r* = 0.359, *p* = 0.020) of medium magnitude. There association between Future Positive Thinking in the Abstract Condition and Trait Rumination was small and non-significant (*r* = 0.051, *p* >0.05). In the Concrete Condition, the associations between trait rumination and Negative and Positive Future Thinking were small and not significant (*r* = 0.155, *p*>0.05; and *r* = -0.072, *p*>0.05, respectively). Collapsing across the two conditions, a small to medium association was found between trait rumination and Negative Future Thinking (*r* = 0.272, *p* = 0.015) but not Positive Future Thinking (*r* = 0.003, *p*>0.05).

## Study 2 Conclusions

In line with hypotheses Study 2 demonstrates that adolescents who are engaged in an abstract mode of processing generate significantly more negative future events than those who engage in a concrete processing style. State mood did not account for the differences observed. In contrast to hypotheses, there were no differences between high and low depression symptom groups.

## Discussion

Using an experimental design with a sample of young adolescents, we found that inducing the abstract processing mode that is characteristic of depressive rumination is associated with poorer social problem-solving (Study 1) and more negative, but not fewer positive, future thoughts (Study 2), compared to inducing a concrete processing mode.

These findings of reduced social problem-solving performance and greater negative future thinking during abstract processing in adolescents are consistent with findings in adults. It suggests that this processing mode, associated with reduced situational and contextual specificity, may be important in influencing the consequences of rumination in adolescents, as it is in adults. Findings from correlational analysis are also consistent with this conclusion. Our finding that changes in state mood did not account for the observed condition effects in either study speaks against the possible explanation that it is due to changes in affect.

Our finding in Study 2 that processing mode did not influence the number of positive future events recalled is in line with other studies with adolescents. For example, increased negative future thinking was found in adolescents with depression in the absence of reduced positive thinking (Miles et al., 2004) (Miles et al., 2004). Similarly, de Jong-Meye(de Jong-Meyer et al., 2007)r and colleagues (2007) reported that completion of a negative mood induction increased negative future, but not reduced positive, thinking in adolescents. The research to date suggests that whilst depressed adolescents experience increased expectation of negative future events following their experience of negative life events due to their projection of autobiographical memory to the future (Tulving(Tulving, 2002), 2002), depressed adolescents do not experience reduced expectation for positive events in their future as potentially, in comparison to adults, they perceive aspects of their future as more changeable and controllable.

We were surprised to find that the effects of manipulating processing mode on problem-solving and negative future thinking held for adolescents scoring at both the high and the low end of the depression measure. In studies with adults, the deleterious effects of abstract processing have only been documented in dysphoric or depressed individuals (Nolen-Hoeksema et al., 2008). It has been suggested that depressed affect is one of the necessary setting conditions under which rumination causes impairment (Watkins, 2008). However, it is noteworthy that our findings are concordant with other studies that have been carried out with adolescents (Park et al., 2004).

It is interesting to consider the possible causes of this contrasting finding in adolescents, assuming it is replicated in future studies. It could be that adolescents are more amenable to brief training procedures than adults because thinking styles may be less ingrained or habitual. A related explanation is that young people are more vulnerable to the negative consequences of ruminative thinking compared to adults.

For example, with regards to negative future thinking, direct comparison of the number of negative future events generated during abstract processing in the current study with findings of Lavender & Watkins (2004) is supportive of this: depressed and non-depressed adult participants generated a mean total of 10.36 and 7.73 respectively and high depression and low depression adolescents generated an average of 12.36 and 10.75 negative future events respectively.

Whilst no group differences were observed between the conditions in either study, the high depression group endorsed significantly higher trait rumination compared to the low depression group (as measured by the CRSQ-R; 19.22 and 16.24 for the High Depression Groups in Study 1 and 2 respectively and 3.27 and 2.23 for the Low Groups for Study 1 and 2). Furthermore, we found trait rumination was associated poorer social problem-solving and more negative future thinking in the Abstract Condition for both studies, indicating that individual differences in habitual rumination may be associated with the outcomes of interest. If adolescents who are experiencing more depressive symptoms are more prone to engage in rumination in general, it is likely that they will also be more likely to adopt an abstract processing mode in the face of difficult feelings and emotions with (abstract) rumination^4^. The findings from our studies suggest that this will exacerbate symptoms, because it will impede problem-solving and bring to mind more negative thoughts about the future. In future, studies using experience momentary sampling could confirm whether more depressed adolescents experience more abstract (vs. concrete) thoughts.

The studies have a number of limitations that warrant mention. With regards Study 1, the MEPS data was coded by two trained researchers showing good interrater reliability, however only one was blind to condition. Future studies with both raters blinded would reduce the potential for bias. It could be argued that the MEPS does not adequately map onto ‘real-life’ social problem-solving. However the measure has construct validity and provides a far more ecologically valid measure than the self-report questionnaires that are typically used to assess problem-solving. Future studies replicating Study 1 findings with other laboratory measures of social problem-solving, such as the Social Problem-Solving Skills Test (Nock & Mendes, 2008) would be valuable. With regards Study 2, a self-report measure of future thinking was used and it is possible that the observed differences in future thinking may reflect particular strategies employed by adolescents (for example, to alleviate negative affect). The use of an implicit response latency task may overcome this limitation. However, given the dual-task design of the study it was necessary to select a task that was not burdensome and allowed young people to continue to engage in the allocated processing style at the same time. The absence of a non-active control group in the studies limits the conclusions that can be drawn regarding the relative contribution of abstract and concrete processing to the observed effects.

We recruited an analogue population scoring in the upper and lower quartiles of a depression measure not a clinical population and so caution should be taken when considering the relevance of these findings to depressed adolescents. However, the average score of those in the High Symptoms Groups (M = 29.44 and 30.51 for Study 1 and 2 respectively) was in the suggested clinical range (scores above 29 have been found to optimally discriminate youth with major depression (Burleson Daviss et al., 2006)), and participants were only included in the study if they endorsed a consistent MFQ score at screening and experimental testing. Taken together, it seems likely that those included in the High Symptoms Group were experiencing a high and stable level of depressive symptoms. It would be informative to carry out the study with a clinical population. Should the findings be replicated, they would point to the value of reducing abstract processing in cognitive behavioural interventions for adolescent depression. The benefit of conducting these studies with a group of high depression scorers (rather than a clinical population) is that it offers an insight into the mechanisms by which rumination may operate in a high-risk population. Adolescence is a time when the incidence of depression rises sharply (Keyes & Platt, 2024), and so delineating risk as well as maintenance mechanisms should be a priority during this period. In this vein, the findings suggest that rumination may be an appropriate target for prevention programs, for example via training individuals to process information in a more concrete style.

Our findings provide evidence that the processing-mode theory of rumination (Moberly & Watkins, 2006; Watkins, 2004) may be useful to invoke in adolescents as well as in adults. The account suggests that the negative emotional and cognitive consequences of rumination are in part due to the abstract mode of processing associated with this thinking (Roberts et al., 2020). The abstract mode of processing involves general, higher-level representations that convey the basic idea rather than the specifics. This may make it difficult to specify a problem or to generate detailed solutions to social problems, as well as priming negative cognitions including future thoughts. Concrete processing, on the other hand, involves representations that include the details and specifics of events and situations, for example, how that particular event came about, where it happened, who was there, what they did.

Our findings point to the potential value of targeting abstract processing, via enhancing a concrete mindset (Topper et al., 2017), amongst young people experiencing high levels of depression symptoms.

## Supplementary Material

Supplementary File

## Figures and Tables

**Table 1:** Study 1 Participant Details.

	High MFQ Group		Low MFQ Group	
	Abstract	Concrete	Abstract	Concrete
N [Female]	22 [12]	28 [17]	23 [6]	22 [10]
Mean (SD, range)				
Age (years)	13.14 [1.46, 11- 15]	12.75 [1.29, 11- 15]	12.78 [1.38, 11- 15]	13.00 [1.27, 11- 15]
Depression (MFQ)	31.00 [11.84, 13- 59]	28.87 [11.21, 14- 48]	1.67 [1.64, 0-5]	1.85 [2.29, 0-5]
Anxiety (SCARED)	30.09 [13.36, 4- 56]	30.37 [13.77, 3- 58]	6.50 [4.93, 0-17]	7.24 [6.17, 0-17]
Rumination (CRSQ-R)	17.94 [9.94, 1-37]	17.66 [8.50, 3-39]	2.88 [3.15, 0-13]	2.71 [2.97, 0-14]
Attentional Control (ACS)	49.62 [8.03, 37- 62]	47.94 [8.16, 29- 61]	58.83 [6.49, 51- 77]	56.64 [5.93, 45- 68]
Verbal Fluency	50.71 [13.76, 25- 87]	50.04 [11.50, 28- 72]	53.17 [12.22, 36- 93]	50.93 [12.25, 29- 79]

**Table 2:** Study 2 Participant Details.

	High MFQ Group		Low MFQ Group	
	Abstract	Concrete	Abstract	Concrete
N [Female]	23 [17]	20 [12]	19 [9]	19 [5]
Mean [SD, range]				
Age (years)	12.78 [1.28, 11- 15]	13.00 [1.26, 11- 15]	12.68 [1.25, 11- 14]	12.95 [1.31 11-15]
Depression (MFQ)	30.74 [12.36, 16- 45]	30.22 [11.58, 15- 50]	1.67 [1.88, 0-5]	1.83 [2.23, 0-5]
Anxiety (SCARED)	31.12 [14.17, 9- 59]	29.50 [13.26, 3- 52]	6.50 [4.60, 0-20]	7.38 [7.42, 0-29]
Rumination (CRSQ-R)	16.55 [10.04, 1- 34]	15.88 [7.51, 2-34]	2.64 [2.95, 0-9]	1.80 [2.07, 0-8]
Hopelessness (HSC)	6.95 [2.17, 3-11]	6.58 [2.14, 3-10]	5.13 [1.06, 3-7]	5.34 [0.89, 4-8]
Attentional Control (ACS)	48.86 [7.81, 37- 63]	49.85 [8.28, 35- 68]	57.21 [5.73, 48- 71]	56.94 [5.57, 50- 69]
Verbal Fluency	50.70 [11.03, 36- 73]	48.90 [11.72, 34- 78]	48.58 [10.15, 27- 64]	48.47 [13.16, 30- 75]

**Table 3:** Study 2 Mean scores on the negative and positive future thinking task.

	Low MFQ Group		High MFQ Group	
	Abstract	Concrete	Abstract	Concrete
Negative Future Thinking	10.75 (3.80)	6.58 (3.14)	12.36 (4.56)	7.22 (2.83)
Positive Future Thinking	15.19 (6.19)	13.52 (4.65)	13.00 (4.87)	13.94 (4.77)

## Footnotes

1 See Supplementary Materials for details of piloting.

2 The scenarios were developed during piloting and on the basis of feedback from a small group of adolescents. The scenarios were acceptable to the participants and they were able to engage with the practice scenarios during the experimental training phase.

3 Please see Supplementary Materials for full results (and for all other instances in the manuscript where full results which have been presented in this simplified format).

4 We note that our measure of trait rumination, the CRSQ, does not explicitly measure abstract thinking but the brooding subscale is arguably capturing overlapping aspects of the process. Future studies could more precisely investigate this.
