# Supplementary material for: Why is rumination unhelpful in adolescents? Two studies examining the causal role of abstract processing
Source: J Affect Disord. Author manuscript; Available in PMC 2026 Jan 30. (PMC7618692; doi:10.1016/j.jad.2025.03.058)
Supplement: Supplementary File [file EMS211716-supplement-Supplementary_File.docx]

**SUPPLEMENTARY MATERIAL**

**‘****Why is rumination unhelpful in adolescents? Two studies examining the causal role of abstract processing.’**

**Pilot**

*Pilot Procedure*

A pilot study was conducted prior to the recruitment and testing of the final sample. Initially the processing style induction materials were piloted with a sample of 15 non-clinical adults. Following adaptation, piloting of the processing style inductions was undertaken with a further 10 non-clinical adults and seven adults with symptoms of depression (scoring above recommended clinical cut-off on the Hospital Anxiety and Depression Scale; Zigmond & Snaith, 1983). Piloting of the processing style inductions and the two experimental tasks was then undertaken with three non-clinical young people (two girls and one boy, aged 11-16 years, mapping onto the sample included in the final study) to ensure that the materials were developmentally appropriate.

The adaptations are outlined below.

*Processing Style inductions*

Experimental processing style inductions were developed based on the instructions outlined by Watkins and Moberly (2008) and feedback from piloting.

Following randomisation, participants were trained to engage in a ruminative or concrete processing style. Participants were introduced to the concepts of concrete or ruminative thinking and instructed to engage in a processing style characterised by ‘how’ and ‘why’ type questions, respectively (see Table). Participants in the concrete condition were shown seven ‘how’ type questions focussing on concrete and sensory details of the situation and discussed how people, when thinking in this way, may imagine the details the scenario whilst adopting a first-person perspective. Participants in the ruminative condition were shown seven ‘why’ type questions focusing on the causes, meanings and consequences of the situation.

Following this introduction, participants in both conditions were trained in the allocated processing style using two imaginary scenarios. On the basis of feedback from the adult pilot sample, participants were provided with full task instructions before the scenarios were outlined to prevent any disruption of further instruction on the processing style induction. Where necessary, further feedback and explanation was given to ensure that participants understood the instructions of the required processing style before training started.

**Table: Questions used in the introduction of the experimental processing styles**

| **Concrete ‘How’ questions** | **Ruminative ‘why’ questions** |
| --- | --- |
| How did this happen?  What did I notice?  What is the sequence of events leading up to this point?  How can I understand this?  How can I fix this?  What is the first step toward solving this problem?  How can I decide what to do next? | Why did this happen to me?  What are the consequences of this?  What will happen because of this?  What will others think of me?  What does this mean about me?  What have I done to deserve this?  Why do things like this keep happening to me? |

When it was established that the participant understood the task, they were read the first imaginary scenario and instructed to spend two minutes imagining themselves in the scenario whilst answering, in their mind, questions presented on a prompt card. The thematic content of the two scenarios was developed using qualitative feedback from three adolescents during piloting. The two scenarios were the same in the thinking style induction training in both experimental conditions. Participants in the ruminative condition were presented with ‘why’ type questions and those in the concreteness condition were presented with ‘how’ type questions and instructed to work through the questions in their mind for two minutes. Participants were then briefly reminded of the task instructions before being read the second imaginary scenario and handed a prompt card. Following feedback from the piloting sample, the induction questions were provided on prompt cards visible for the two minute induction period for each scenario and were tailored to the individual situations.

Prior to the processing style inductions, participants were asked to identify two situations within the last week that had made them feel sad, that had been on their mind a lot and that they were comfortable to disclose to the researcher. Following feedback from non-clinical adults during piloting, example situations (eg: ‘this could be an argument with friends or family or a difficulty at school’) were provided by the researcher to prompt participants who may have difficulties identifying such situations. Following completion of the thinking style training, all participants were asked to apply the trained thinking style to each of their own personal situations. Participants were reminded of the situations they had outlined and were asked to think back to a time that the situation had been particularly pertinent. They were then instructed to remember that time as vividly as possible, focusing on cognitions, affect and somatic sensations. Participants were reminded of the task instructions, provided with a prompt card and instructed to think about the situation for two minutes in the processing style in which they had been trained. Participants were instructed to continue thinking in the style in which they had been trained, keeping in mind either ‘how’ or ‘why’ questions whilst completing one of two experimental tasks according to counter balanced allocation of presentation order. The induction was then repeated for the second personal situation, participants were again instructed to continue thinking in the style in which they had been trained, and the second experimental task was completed.

*MEPS*

Scenarios were developed with young people (n=5, aged 11-16 years). This involved a one off ‘brainstorming’ meeting with the young people to identify relevant scenarios. The researchers brought example scenarios to support the process. Scenarios were then piloted with the (n=3) young pilot participants. They reported that the scenarios were understandable and relevant and they were able to engage with the practice scenarios during the experimental training phase.

*Future Thinking Task*

Piloting indicated that young people were able to complete the task and identify future events across all time periods and no changes were made.

**Study 1 Additional Results**

*Baseline Characteristics*

For age, there were no significant differences across group (*F*(1, 91) = 0.002, *p*=.968), condition (*F*(1, 91) = 0.03, *p*=.872), or interaction (*F*(1, 91) = 1.03, *p*=.314). For MFQ scores, there were no significant differences across condition (*F*(1, 90) = 0.25, *p*=.617), or interaction (*F*(1, 90) = 0.29, *p*=.348). For SCARED anxiety scores, there were no significant differences across condition (*F*(1, 89) = 0.44, *p*=.507), or interaction (*F*(1, 89) = 0.001, *p*=.970). For trait rumination scores (CRSQ), there were no significant differences across condition (*F*(1, 88) = 0.15, *p*=.700), or interaction (*F*(1, 88) = 0.19, *p*=.666). For verbal fluency, no significant differences were found across groups (*F*(1, 91) = 2.46, *p*=.120), conditions (*F*(1, 91) = 0.50, *p*=.483), or interaction (*F*(1, 91) = 0.57, *p*=.454).

**Study 2 Additional Results**

*Baseline Characteristics*

For age, there were no significant differences across group (*F*(1, 77) = 0.02, *p*=.902), condition (*F*(1, 77) = 0.94, *p*=.335), or interaction (*F*(1, 77) < 0.001, *p*=.991). For MFQ scores, there were no significant differences across condition (*F*(1, 77) = 0.07, *p*=.798), or interaction (*F*(1, 77) = 0.07, *p*=.786). For SCARED anxiety scores, there were no significant differences across condition (*F*(1, 76) = 0.02, *p*=.877), or interaction (*F*(1, 76) = 0.18, *p*=.670). For trait rumination scores (CRSQ), there were no significant differences across condition (*F*(1, 76) = 0.08, *p*=.773), or interaction (*F*(1, 76) = 0.13, *p*=.719). For verbal fluency, no significant differences were found across groups (*F*(1, 77) = 0.24, *p*=.623), conditions (*F*(1, 77) = 0.14, *p*=.713), or interaction (*F*(1, 77) = 0.11, *p*=.743).
